# Editorial: Multi-Target-Directed Ligands (MTDL) as Challenging Research Tools in Drug Discovery: From Design to Pharmacological Evaluation

**DOI:** 10.3389/fchem.2019.00071

**Published:** 2019-02-18

**Authors:** Stefano Alcaro, Maria Laura Bolognesi, Alfonso T. García-Sosa, Simona Rapposelli

**Affiliations:** ^1^Net4Science Academic Spin-Off, Università “Magna Græcia” di Catanzaro, Campus Universitario “S. Venuta”, Catanzaro, Italy; ^2^Department of Pharmacy and Biotechnology, Università di Bologna, Bologna, Italy; ^3^Department of Molecular Technology, Institute of Chemistry, University of Tartu, Tartu, Estonia; ^4^Department of Pharmacy, Università di Pisa, Pisa, Italy

**Keywords:** multi-target paradigms, multi-target molecules, multidisciplinary cooperation, drug discovery, chemotheca, compliance, rational drug design

The philosophy “one molecule—one target—one disease” was the dominant approach in medicinal chemistry for several decades up till the end of the twentieth century. This strategy was based on the identification and optimization of small chemical entities able to recognize specifically one target, believed to be fully responsible for a certain disease. The aim of the “one drug-one target” approach was to find bioactive compounds endowed with a limited risk of off-target properties, many times responsible for drug side-effects. This philosophy started to change in the last 20 years due to the emergent growing awareness that drugs designed to act on individual molecular targets are usually inadequate for multigenic diseases such as cancer, neurodegenerative, and infectious diseases (Ramsay et al., 2018). System biology and omic sciences recently enriched the knowledge of the complex world underlying their pathogenesis, depicting the importance of networked signaling pathways and suggesting multi-target therapeutics as potentially more advantageous than mono-therapies. Nowadays, so called “drug-cocktails” are often the only available approaches to pharmacologically treat many of these pathologies, with concerns related to drug-drug interactions as well as to patients' compliance. Hence, it is appropriate and relevant to search for novel bioactive compounds able to combine in one molecule multi-target properties, according to the paradigm of “network pharmacology” (Hopkins, 2008).

Therefore, a special issue dedicated to collect research activities from critical areas for the development of novel multi-target-directed ligands (MTDL) through the close cooperation among pharmacologists, biochemists, medicinal chemists, and toxicologists, has been proposed. This research topic should collect new approaches developed to overcome the main issues faced by medicinal chemists in the design, synthesis, and biological evaluation of these promising, but extremely challenging, new chemical entities. The first is the identification of new MTDL derived either from natural sources or synthetic procedures. The second is related to the-state-of-the-art biological and biophysical tests particularly suited to rapidly explore a multi-target profile. The third concerns the creation of chemoinformatic tools such as a chemical database for the collection and management of multi-target agents. The fourth is dedicated to advanced methods for the *in silico* estimation of multi-target ligands by means of docking and virtual screening tools. The contribution submitted by Oliveira et al. deals with the design and synthesis of new multitarget compounds that target mitochondrial oxidative stress (OS) and restore cholinergic transmission. The new molecules show favorable toxicological profile, neuroprotective activity, and drug-like properties, thus suggesting a fine blood-brain barrier (BBB) permeability. All together, these results indicate that the anticholinesterase inhibition coupled with antioxidant properties is an effective therapeutic strategy for Alzheimer's disease (Oliveira et al.) Another contribution was submitted by Elshaflu et al. In this case, the proposed ligands were able to inhibit monoaminooxidase (MAO) isoforms based on the isosteric replacement (S −> Se) within the (1,3-thiazol-2-yl)hydrazine scaffold. This chemical manipulation induced an improvement of antioxidant properties. Moreover, *in-silico* calculations of ADME properties showed good pharmacokinetic profiles of some compounds investigated. The manuscript submitted by Albreht et al. deals with the clarification of the mechanism of action of MTDLs containing the propargylamine as the reactive moiety toward MAO cofactor. In this paper, a rational design of efficient new generation drugs for the treatment of neurodegenerative and neuropsychiatric disorders is suggested (Albreht et al.). The manuscript from Koch et al. focused on MAO inhibition as co-target together with human A_1_ and A_2A_ adenosine receptors (ARs). The study deals with the synthesis and *in vitro* evaluation of novel annelated xanthine derivatives. The multitarget activity of such compounds paves the way to a potential application for the treatment of neurodegenerative diseases, in particular Parkinson's disease (Koch et al.). The manuscript submitted by Ortuso et al. explicitly discussed the creation of a chemical database and the implementation of the Chemotheca platform, the networking tool specifically useful for speeding up the multi-target drug discovery process (Ortuso et al.). In another contribution, by Yosipof et al. a chemical database was also used to carry out the proposed *in silico* work (Yosipof et al.). Machine learning methods were applied in a multi-target fashion to distinguish drug and non-drugs for three different classes of compounds. The ability to use such tools for the identification of interesting trends opens up new opportunities for understanding the factors affecting drug performance and for designing new drugs. Another interesting manuscript collected in this research topic is that by Roman et al. which discussed *in silico* approaches to predict also antitarget and physicochemical profiles of (*S*)-blebbistatin, the best-known myosin II ATPase inhibitor, and a series of analogs (Roman et al.). This paper is a good example concerning the use of *in silico* techniques which should be useful for accelerating the discovery of new molecules with appropriate target and antitarget profiles. Finally, the paper by Pagano et al. is an interesting publication related to multi-targeting agents able to recognize DNA in multiple non-canonical conformations, typically folding as a G-quadruplex motif (Pagano et al.).

In conclusion, this research topic launched in January 2018 and closed in April 2018 has gathered contributions from design, synthesis, and biological evaluation of multi-target molecules. This research topic has covered most of the competencies encompassed in such a modern and stimulating field of drug discovery. We are aware that it is far more complex than what this research topic can capture, but we wish we can contribute to add pieces to the puzzle of rational and effective multi-target drug discovery.

## Author Contributions

SA managed the structure and the submission of the manuscript. MB supervised the multi-target contents and definitions into the manuscript. AG-S controlled the reference analysis. SR organized the manuscript revision and the keywords' definition.

### Conflict of Interest Statement

The authors declare that the research was conducted in the absence of any commercial or financial relationships that could be construed as a potential conflict of interest.
